# CD4+CD25highCD127low Regulatory T Cells in Peripheral Blood Are Not an Independent Factor for Chronic Graft-versus-Host Disease after Allogeneic Stem Cell Transplantation

**DOI:** 10.1100/2012/606839

**Published:** 2012-05-01

**Authors:** Jolanta B. Perz, Selma Gürel, Stefan O. Schonland, Ute Hegenbart, Anthony D. Ho, Peter Dreger

**Affiliations:** ^1^Department of Internal Medicine-Hematology, Oncology and Rheumatology, University Hospital Heidelberg, 69120 Heidelberg, Germany; ^2^Bone Marrow Transplantation Unit, Department of Internal Medicine-Hematology, Oncology and Rheumatology, University Hospital Heidelberg, 69120 Heidelberg, Germany

## Abstract

*Background*. The therapeutic efficacy of allogeneic hemopoietic stem cell transplantation (HSCT) largely relies on the graft-versus-leukemia (GVL) effect. Uncontrolled graft-versus-host disease (GVHD) is a feared complication of HSCT. Regulatory T cells (Treg) are a subset of CD4+ T-helper cells believed to maintain tolerance after HSCT. It remains unclear whether low peripheral blood Treg have an impact on the risk for acute (aGVHD) and chronic GVHD (cGVHD). *Methods*. In this paper we enumerated the CD4+CD25highCD127low Treg in the peripheral blood of 84 patients after at least 150 days from HSCT and in 20 healthy age-matched controls. *Results*. Although similar mean lymphocyte counts were found in patients and controls, CD3+CD4+ T-cell counts were significantly lower in patients. Patients also had significantly lower Treg percentages among lymphocytes as compared to controls. Patients with cGVHD had even higher percentages of Treg if compared to patients without cGVHD. In multivariate analysis, Treg percentages were not an independent factor for cGVHD. *Conclusions*. This paper did not show a relation between deficient peripheral blood Treg and cGVHD, therefore cGVHD does not seem to occur as a result of peripheral Treg paucity.

## 1. Introduction

Allogeneic hemopoietic stem cell transplantation (HSCT) is a well-established treatment procedure with the potential to cure a variety of hematological diseases. The graft-versus-leukemia (GVL) effect exerted by donor T cells can largely contribute to its efficacy, but the donor T-cell-mediated graft-versus-host disease (GVHD) is a feared complication. The challenge of allogeneic HSCT is to find a balance between beneficial and harmful T-cell effects. Naturally arising regulatory T cells (Treg), a subset of CD4+ T-helper cells expressing high levels of the interleukin-2 receptor *α*-chain CD25 and the transcription factor forkhead box P3 (FoxP3) [1], have the ability to suppress aberrant immune responses, to regulate peripheral T-cell homeostasis, and to maintain self-tolerance [2–4]. Treg are believed to maintain tolerance after solid organ transplantation and after HSCT [5]. Studies using murine transplantation models showed that allogeneic Treg promote engraftment, allow long-term tolerance, and decrease incidence and severity of GVHD without abrogating the beneficial GVL effect [6–13]. Human studies investigating the impact of Treg on complications after HSCT have so far generated rather conflicting results.

Generally CD4+CD25high T cells, which coexpress FoxP3 are accepted as Treg [1, 3, 4]; however, FoxP3 is an intracellular marker. Studies of Treg were hampered by the lack of a suitable cell-surface marker apart from CD25, until Liu et al. demonstrated that the downregulation of the *α*-chain of the interleukin-7 receptor (CD127) on the majority of the FoxP3 positive CD4+ T cells distinguishes Treg from activated T cells [14]. As CD127 is a cell surface marker, the CD4+CD25highCD127low phenotype allows for consistent identification of human Treg in quantitative studies as well as for enrichment of live Treg for functional analyses or expansion in vitro [14, 15]. As determined by functional assays, CD4+CD25highCD127low Treg show a suppressive effect in mixed lymphocyte culture studies [16, 17].

Several human studies have investigated the impact of Treg on the complications after HSCT. Zorn et al. found lower frequencies of CD4+CD25high Treg in the peripheral blood of patients suffering from chronic GVHD (cGVHD) compared to patients without cGVHD and to healthy individuals; however, without differences in functional studies [24]. Nadal et al. found higher frequencies of CD4+CD25high Treg in the peripheral blood of patients with chronic myeloid leukemia (CML) more than 18 months after allogeneic HSCT compared to normal controls and to newly diagnosed CML patients [18]. The study did not find a difference in Treg levels between patients with or without cGVHD; however, patients with high Treg levels showed evidence of disease relapse, thus an inhibition of the GVL effect by Treg was suggested [18]. CD4+CD25highFOXP3+ Treg were postulated to have a diagnostic and prognostic value as a biomarker for acute GVHD (aGVHD) [19]. Ukena et al. analyzed the recovery of CD4+CD25highCD127low Treg after HSCT and found that numerically deficient Treg were associated with the development of aGVHD but not cGVHD [20].

## 2. Objective

Until now the mechanisms of Treg recovery and their suppressive activity in individuals after allogeneic HSCT are not well understood. In this study we performed a cross-sectional analysis of the lymphocyte subpopulations and particularly of the CD4+CD25highCD127low Treg in the peripheral blood in 84 patients after at least 150 days from allogeneic HSCT and in 20 healthy age-matched controls. The aim of the study was to investigate the immunoreconstitution, especially the Treg reconstitution after HSCT, to find factors influencing Treg recovery and to assess the impact of peripheral blood Treg on the development of cGVHD.

## 3. Material and Methods

### 3.1. Subjects

Peripheral blood samples were routinely collected after written informed consent was obtained in accordance with the Declaration of Helsinki of 1975, as revised in 2000, from 84 consecutive patients, who had undergone allogeneic HSCT for hematological diseases at the University Hospital in Heidelberg and from 20 healthy controls. The local ethics committee at the University of Heidelberg had approved sample collection. The median age of controls (12 female, 8 male) was 42 years (25–62), similarly the median age of patients (50 male, 34 female) was 46 years (19–67).

### 3.2. Stem Cell Transplantation Procedure

Details on transplantation procedure and donor matching are summarized in Table 1. Patients undergoing unrelated donor transplantation received in vivo T-cell depletion with antithymocyte globulin (ATG Fresenius) at total dose of 30–60 mg/kg. Patients with standard conditioning before HSCT received cyclosporine A and short course of methotrexate on days +1, +3, and +6 whereas patients with reduced intensity conditioning (RIC) received cyclosporine A and mycophenolate mofetil for GVHD prophylaxis. At the time of investigation, the median time from transplantation was 533 days (range 150–3606 days) and all patients attended the outpatient unit. Patients were subdivided in three cohorts depending on the time-point of investigation after HSCT: patients in cohort 1 (*n* = 26) had the assessment within the timeframe of 150–360 (median 244) days after HSCT, patients in cohort 2 (*n* = 22) within the timeframe of 360–720 (median 510) days after HSCT, and patients in cohort 3 (*n* = 36) after more than 720 (median 1182, range 737–3606) days after HSCT. This cohort analysis was performed in order to approximate the immunoreconstitution at the different time periods from HSCT.

At the time of the study 31 patients (37%) were on immunosuppressive treatment or had stopped immunosuppressives less than 2 weeks prior to the study (9 cyclosporin A, 2 tacrolimus, 5 mycophenolate mofetil, 3 steroids, 7 combination of calcineurin inhibitors with steroids, and 5 combination of calcineurin inhibitors with mycophenolate mofetil). Molecular chimerism studies using short tandem repeats (STR) amplification of nine loci performed on the mononuclear cell fraction in the bone marrow showed at least a >95% donor chimerism in all 84 patients. AGVHD and cGVHD were assessed and graded according to standard criteria [21, 22]. At the time of the study 38 patients did not have clinical signs of cGVHD whereas 46 patients (55%) presented signs of cGVHD and 27 patients (32% of all) suffered from extensive cGVHD.

### 3.3. Flow Cytometry

#### 3.3.1. CD3+CD4+CD25highCD127low Treg Staining

The enumeration of the lymphocyte subpopulations including the CD4+CD25highCD127low Treg was performed by flow cytometry from fresh peripheral blood samples in ethylenediaminetetraacetic acid (EDTA). A blood count was obtained from each sample and subsequently a staining with appropriate combinations of the following monoclonal antibodies: fluorescein isothiocyanate- (FITC-) conjugated anti-CD25 (Beckman Coulter, Krefeld, Germany) and anti-CD8 (Becton Dickinson (BD) Pharmingen, Heidelberg Germany); phycoerythrin- (PE-) conjugated anti-CD127 (BD Pharmingen) and anti-CD19 (BD); peridiumchlorophyllprotein-indopentamethicynine PerCP-Cy5.5-conjugated anti-CD4 (BD); allophycocyanin- (APC-) conjugated anti-CD56 (BD); phycoerythrin-cyanine- (PE-Cy7-) conjugated CD3 (Caltag Laboratories, Botolph Claydon, UK) as well as IgG isotype controls. Briefly, 100 *μ*l of undiluted sample containing between 0.5–1 × 10^6^ nucleated cells were incubated with a combination of monoclonal antibodies for 15 minutes in darkness at room temperature. 1 mL of FACS wash and lyse solution (Becton Dickinson BD) diluted 1 : 10 in distilled water were added to each tube and after vortexing another incubation for 15 minutes in darkness at room temperature was performed. 

**Figure 1:**
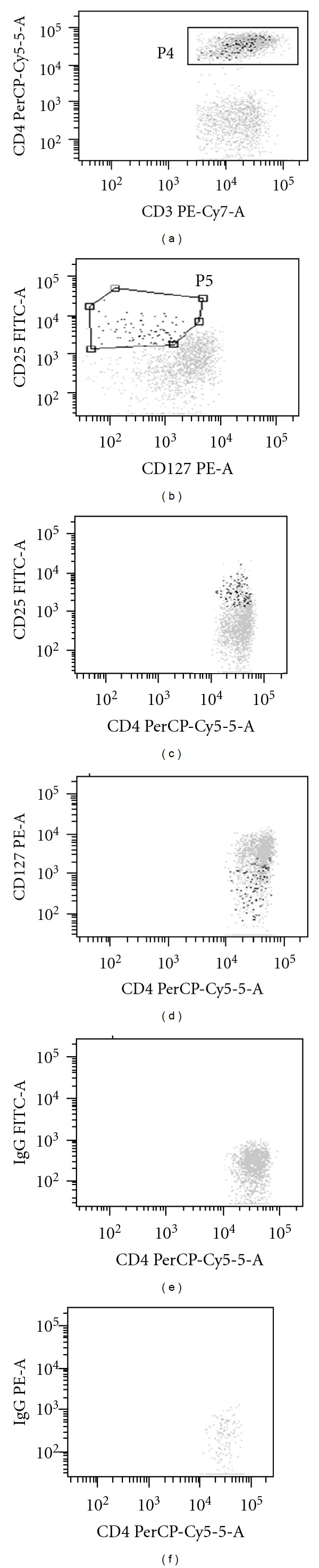
Treg gating strategy. Representative bivariate dot plots illustrating gating strategy used to enumerate lymphocytes with the CD3+CD4+CD25highCD127low phenotype (Treg) in the same blood sample. The settings showed here were used for the analysis of all peripheral blood samples. Events corresponding to Treg are shown as black dots, events corresponding to other lymphocytes are shown as gray dots in all bivariate dot plots. (a) Gate shows CD3+CD4+ events corresponding to T-helper lymphocytes; (b) CD25highCD127low events corresponding to Treg are displayed in the gate; (c) bivariate dot plot illustrating the CD4+CD25high phenotype pattern of Treg; (d) bivariate dot plot illustrating the CD4+CD127low phenotype pattern of Treg: (e) and (f) bivariate dot plots showing appropriate isotype controls. CD: cluster of differentiation.

Cells were then centrifuged (5 minutes at 1300 U/min). The last two steps were repeated and the labeled cells were finally resuspended in 0.5 mL/tube of phosphate buffered saline. Antigen expression was analysed on an FACS Canto flow cytometer (Becton Dickinson, San Jose, CA, USA). Data acquisition was performed with at least 50 000 cells per sample using BD Diva software (Becton Dickinson). Data were analysed with Infinicyt 1.0 software (Cytognos, Salamanca, Spain). Frequencies of T-cell subpopulations were calculated as percentages of positive cells in the total lymphocyte gate.  Figure 1 shows the gating strategy used to define the Treg population. Absolute cell numbers for the T-cell subpopulations were calculated from the leukocyte and lymphocyte counts given by the full blood count performed on the same sample. 

#### 3.3.2. CD3+CD4+CD25highFoxP3+ Treg Staining

Samples were incubated with surface antibodies first, cells were then fixed and permeabilized using the Human FoxP3 Buffer Set (BD Pharmingen) and incubated with PE-conjugated anti-FoxP3 (clone 259D/C7, BD Pharmingen) following the manufacturer's staining procedure recommendations. Data were acquired and analysed as described above. In 20 healthy controls a positive correlation was found between the percentages of CD3+CD4+CD25highCD127low and CD3+CD4+CD25highFoxP3+ lymphocytes (*r* = 0.89, *P* = 0.002).

### 3.4. Statistical Analysis

Data are presented as median and range or depicted as Box-Whisker plots [23]. Clinical characteristics that may influence variations in lymphocyte subpopulations were compared using Mann-Whitney *U* test for 2 groups or Kruskal-Wallis test for three or more groups. Fisher's exact test was used to compare categorical variables in the groups of patients with and without cGVHD. Correlation was calculated using a rank-based Spearman test. A logistic regression model was applied to analyse the influence of clinical transplant factors on Treg percentages among lymphocytes and on their counts in peripheral blood. Treg values were dichotomized using the median value obtained in the investigated population as a cutoff. A logistic regression model was also applied to analyse the clinical transplant factors and lymphocyte subpopulations between patients with and without cGVHD. Data were analysed using the statistical package StatView for Windows (SAS Institute, Cary, NC, USA). All quoted *P*  values are two sided, with *P* < 0.05 being considered statistically significant.

## 4. Results

### 4.1. Lymphocyte Populations in Patients and Controls

**Figure 2:**
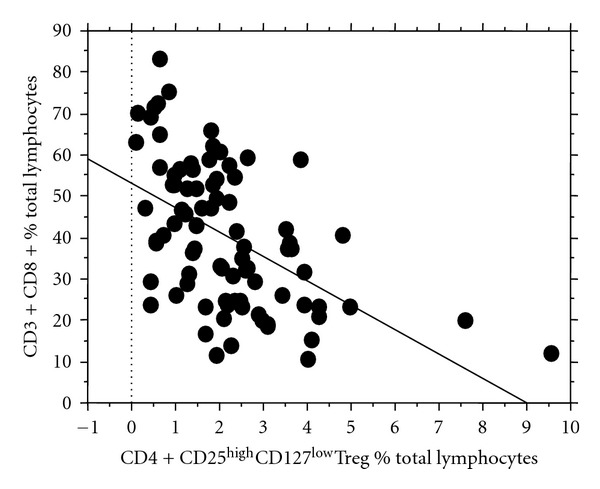
Inverse correlation between T-effector cells and Treg. Percentages of CD3+CD8+ T-effector cells and CD4+CD25highCD127low Treg are inversely correlated (*r* = −0.55, *P* < 0.0001) as shown by the trend line. Dots correspond to individual samples from 84 patients. CD: cluster of differentiation, Treg, regulatory T cells.

Median leukocyte and lymphocyte counts were similar in patients and healthy controls: 6155 × 10^6^/l (1200–14800) versus 6900 × 10^6^/l (4800–9800) and 1377 × 10^6^/l (87–3423.8) versus 1555.9 × 10^6^/l (675.8–2290.8), respectively. CD3+CD4+ T-cell counts were significantly lower in patients as compared to controls: 307.5 × 10^6^/l (32.9–1124.6) versus 808.8 × 10^6^/l (411–1091.4; *P* < 0.001). Percentages of CD4+CD25highCD127low Treg among total lymphocytes and absolute numbers of Treg were significantly lower in patients if compared to controls: 1.9% (0.08–9.5) versus 3.8% (3.4–5.9) and 21.2 × 10^6^/l (1.6–114.7) versus 56.4 × 10^6^/l (39.8–101.4; both *P* < 0.001). Remarkably, no difference was found in percentages of Treg among CD3+CD4+ T-lymphocytes with 8.3% (0.72–23.1) in patients versus 8.0% (5.6–11.9) in controls. On the other hand percentages of CD3+CD8+ T-effector lymphocytes were higher in patients than in controls: 38.9% (10.9–83.6) versus 24.2% (14.4–40.1) (*P* = 0.004) and inversely correlated with Treg percentages (*r*_*s*_ = −0.55, *P* < 0.0001) as shown in Figure 2. Lymphocyte counts, CD3+CD4+ T-cell counts, and Treg counts were positively correlated with the day from HSCT (*r* = 0.25, *P* = 0.02 for lymphocytes, *r* = 0.5, *P* < 0.0001 for CD3+CD4+ T cells and *r* = 0.29, *P* = 0.008 for Treg). Percentages of CD19+ B-lymphocytes and CD3-CD56+ natural killer (NK) cells were similar in patients and controls (16.1% (1.9–55.9) versus 10.6% (8.3–16.4), 2.6% (0.4–18.2) versus 2.9% (1.2–6.7), resp.).

### 4.2. Lymphocyte Populations in Cohorts of Patients Studied at Different Time Periods from HSCT

**Figure 3:**
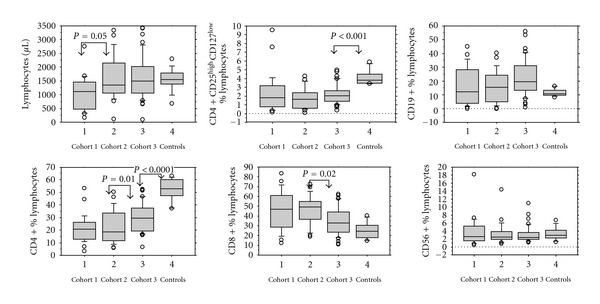
Lymphocyte subpopulations and Treg within different time periods from HSCT. Box plots graphs of lymphocyte subpopulation frequencies in the three patient cohorts and in normal controls. The grafts show the dispersion around the median. The line across each box represents the median for patients in cohort 1 (*n* = 26, median time from HSCT 244 days), in cohort 2 (*n* = 22, median time from HSCT 510 days), in cohort 3 (*n* = 36, median time from HSCT 720 days), and for normal controls (*n* = 20). The bottom edge is the first quartile and the top edge is the third quartile, the error bars represent the dispersion within the 95% confidence interval, the dots represent extreme values. Differences between the groups were explored using the Mann-Whitney *U*-test. Significant differences between the groups are shown above (*P* < 0.05). All other differences between the median values were not significant (*P* > 0.05). CD: cluster of differentiation.

The total leukocyte counts were similar in the cohorts 1, 2, and 3. Lymphocyte and CD3+ T-cell counts were significantly lower in cohort 1 than in the cohorts 2 and 3: 1107.7 × 10^6^/l (161–2744) lymphocytes and 739 × 10^6^/l (92–2333) CD3+ T cells (*P* = 0.05) in cohort 1, but 1348.5 × 10^6^/l (118–3328) lymphocytes and 1029 × 10^6^/l (104–2812) CD3+ T cell in cohort 2 and 1639.7 × 10^6^/l (653–3416) lymphocytes and 1082 × 10^6^/l (512–2320) CD3+ T cells in cohort 3 (*P* < 0.01 for lymphocytes and *P* = 0.05 for CD3+ T cells in cohort 1 if compared to cohort 2 and 3), see Figure 3. CD3+CD4+ T-cell counts were significantly lower in cohort 1 with 149 × 10^6^/l (33–802) than in cohort 2 with 312 × 10^6^/l (46–624; *P* < 0.001). Also, CD3+CD4+ T-cell counts were significantly lower in cohort 2 if compared to cohort 3 (411 × 10^6^/l (214–1124; *P* = 0.03)). The percentages of CD4+CD25highCD127low Treg among total lymphocytes were not different in the three cohorts: 1.8% (0.1–9.5) in cohort 1 versus 1.6% (0.1–4.2) in cohort 2 versus 2.1% (0.7–4.9) in cohort 3 but significantly lower than in controls (3.8% (3.4–5.9; *P* < 0.001)). The percentages of CD4+CD25highCD127low Treg among CD3+CD4+ T cells were not different in the three cohorts: 8.2% (2–18.3) in cohort 1 versus 8.0% (0.9–20.5) in cohort 2 versus 8.4% (1.3–17) in cohort 3 and similar to those in controls (8% (5.6–11.9; *P* < 0.001)). In cohort 3 percentages of CD3+CD8+ T-effector lymphocytes were lower (33%, 14–58) than in the cohorts 1 (47%, 12–83.5) and 2 (49%, 18.5–72.5; *P* = 0.02). As demonstrated in Figure 3 lymphocyte counts reach levels of normal controls already within the second year from HSCT whereas the proportions of CD3+CD4+ T cells and Treg among lymphocytes are lower than in controls even after longer than two years from HSCT.

### 4.3. Analysis of Factors for Treg Expansion after HSCT

The univariate analysis of factors that could potentially impact on Treg reconstitution included the factors: patient age, type of transplant conditioning, donor type (sibling versus unrelated), HLA match, gender match, the use of in vivo T-cell depletion with ATG, CMV serology (negative in recipient and donor versus others), ongoing immunosuppression, and previous acute GVHD and was first performed separately in the three patients cohorts (cohort 1, 2, and 3 as described before) and in the second step in the whole group of *n* = 84 patients.

The analysis showed an impact of negative CMV serology of donor and recipient (*P* = 0.045) on Treg percentages in cohort 2 and an impact of ongoing immunosuppression (*P* = 0.005 for Treg percentages and *P* = 0.02 for Treg counts) in cohort 3 (*n* = 36). The analysis in the whole group of *n* = 84 patients showed a significant correlation between the time from HSCT and Treg counts (*P* = 0.008) and significantly higher Treg percentages in patients with negative CMV serology in both patient and donor (*P* = 0.04). A trend was seen towards higher Treg percentages in patients who received sibling HSCT (*P* = 0.075). The analysis also found significantly higher Treg counts in a transplant setting without the use of ATG (*P* = 0.03). See also Table 2. A logistic regression model using the cutoff value of 1.93% for Treg percentages or the cutoff value of 21.2 × 10^6^/l for Treg counts did not reveal an independent prognostic parameter.

### 4.4. Treg Expansion in Patients with cGvHD and in Patients without cGvHD

**Figure 4:**
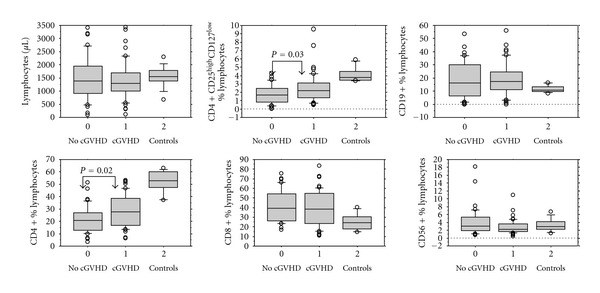
Lymphocyte subpopulations and Treg in patients with cGVHD and in patients without cGVHD. Box plot graphs of lymphocyte subpopulation frequencies in patients and healthy controls. The graphs show the dispersion around the median for patients without cGVHD (*n* = 38), for patients with cGVHD (*n* = 46), and for normal controls (*n* = 20). The line across each box represents the median, the bottom edge the first quartile, and the top edge the third quartile, the error bars represent the dispersion within the 95% confidence interval and the dots represent extreme values. Differences between the groups were explored using the Mann-Whitney *U*-test. Significant differences between the groups are shown above (*P* < 0.05). All other differences between the median values were not significant (*P* > 0.05). CD: cluster of differentiation, Treg: regulatory T cells, and cGVHD, chronic graft versus host disease.

At the time of Treg investigation 46 patients (55%) had clinical signs of cGVHD. Patient with or without cGVHD were not different with respect to age, gender, and transplantation procedure details like type of conditioning, donor type, donor matching, cytomegalovirus matching, gender matching, or blood group matching (see Table 3). As shown in Figure 4 patients with cGVHD had higher CD3+CD4+ T-cell percentages than patients without cGVHD (*P* = 0.02). Treg percentages among total lymphocytes were higher in patients with cGVHD when compared to patients without cGVHD (*P* = 0.03). Treg percentages among CD3+CD4+ T lymphocytes were similar in both patient groups: 8.2% (3.3–23.1) versus 8.5% (0.7–19.5) and also no differences were found with respect to CD3+CD8+ T-effector lymphocytes, CD19+ B cells, and CD3-CD56+ NK cells. In both groups Treg percentages were significantly lower if compared to healthy controls.

 A logistic regression model for cGVHD including the factors donor type and in vivo T-cell depletion with ATG together with CD3+CD4+ T cells did not reveal an independent factor. A similar logistic regression model including the factors donor type and in vivo T-cell depletion with ATG together with Treg percentages or Treg counts did not reveal an independent factor for cGVHD.

## 5. Discussion

In this study we enumerated CD4+CD25highCD127low Treg in order to investigate their reconstitution after allogeneic HSCT and their impact on cGVHD. Similar to the approach used by Zorn et al. we chose to examine the frequency of Treg over total lymphocytes; however, Zorn investigated the CD4+CD25high Treg population whereas we assessed the CD4+CD25highCD127low Treg subset [24]. In contrast, Clark and Nadal investigated the CD4+CD25high Treg population as percentage of CD4+ T-cells [18, 25]. We and others believe, that other than absolute numbers, the frequency measurements reflect the ratio of Treg to the total cells to be regulated and thus represent a more accurate reflection of the Treg function in vivo [24]. Remarkably, the different flow cytometry approaches (CD4+CD25high versus CD4+CD25highCD127low) and the different frequency measurements (Treg as a percentage of CD4+ T cells versus Treg as a percentage of total lymphocytes) may generate conflicting results.

FoxP3 is an intracellular marker and its investigation leads to time- and cost-consuming flow cytometry approaches. Liu et al. demonstrated that by the down regulation of CD127 the FoxP3 positive Treg can be distinguished from activated T cells [14]. The enumeration of the CD4+CD25highCD127low population has since then been chosen to characterize Treg in human studies. Recently Ukena et al. used this approach in patients after allogeneic HSCT [20]. In this study we were able to show a highly significant positive correlation between the CD4+CD25highCD127low and the CD4+FoxP3+ Treg populations in 20 healthy controls and decided to use the CD4+CD25highCD127low Treg enumeration as a cost-effective flow cytometry approach. We have not performed functional tests; however, functional tests in previous studies have confirmed the regulatory capacity of CD4+CD25highCD127low Treg [14, 15]. In contrast to murine studies relatively little is known on reconstitution of T-cell subsets and the impact of Treg on complications after allogeneic HSCT in humans. Rezvani et al. monitored Treg during immune reconstitution in patients with leukemia after T-cell-depleted allogeneic HSCT [26]. Early after HSCT, there was a significant expansion in the CD4+FoxP3+ compartment, on the other hand a low CD4+FoxP3+ Treg count early after HSCT was associated with an increased risk of GVHD [26]. High numbers of CD4+FoxP3+ Treg within the stem cell graft were shown to reduce the risk of aGVHD in HLA matched allogeneic HSCT [26]. Treg content in the graft also predicts the outcome of unrelated donor HSCT [27].

In this cross-sectional study we documented an insufficient recovery of CD4+ T lymphocytes and CD4+CD25highCD127low Treg in patients after at least 150 days from allogeneic HSCT if compared to healthy controls. As expected lymphocyte counts and CD4+ T-cell counts correlated with time from allogeneic HSCT. We showed that also CD4+CD25highCD127low Treg counts correlated with time from allogeneic HSCT. A longitudinal analysis has not been performed in this study, but the cohort analysis of patients at different time-periods from allogeneic HSCT demonstrates that CD4+ T-cell counts are lower than in controls even after more than two years. Treg adequately expand as a proportion of CD4 T-cells already within the first year from HSCT, CD4+ T-cells, however, remain lower than in controls even after 2 years from HSCT and as a consequence Treg counts and percentages among total lymphocytes remain lower than in controls. An inverse correlation was found between the percentages of Treg among total lymphocytes and the percentages of T-effector lymphocytes indicating a physiologic interaction between these two populations. These data confirm the results of previous studies showing that immune reconstitution following allogeneic HSCT is characterizes by a rapid expansion of peripheral CD3+CD8+ T-effector cells and delayed recovery of CD3+CD4+ T-cells [24, 28].

The univariate analysis of factors with potential influence on Treg reconstitution showed that patients with negative CMV serology and CMV negative donors had significantly higher Treg percentages. This fact possibly reflects the impact of CMV virus on reconstitution of CD4+ T-cells and Treg after HSCT. The univariate analysis also showed that patients after longer than 2 years from HSCT and ongoing immunosuppression had lower Treg percentages. A trend towards lower Treg percentages was seen in patients after unrelated donor HSCT, after in vivo T-cell depletion with ATG and in a HLA mismatched transplant setting. The logistic regression analysis of these factors, however, did not reveal an independent factor for Treg reconstitution. This study showed that CD4+CD25highCD127low Treg percentages among CD4+ T-cells are similar to those in healthy controls already within the first year after HSCT, indicating that the recovery of Treg closely follows the CD4+ T-cell recovery. 

CGVHD remains the most frequent complication of allogeneic HSCT occurring in 30% to 70% of long-term survivors and is characterized by a state of profound immunodeficiency and defects in immunologic tolerance [29]. The immune mechanisms leading to diverse clinical manifestations of cGVHD remain largely unknown [30, 31]. Several studies in murine models have demonstrated the critical role of Treg with respect to GVHD control [8–10]. In humans, it remains unclear whether the impairment in peripheral blood Treg after HSCT contributes to the development of GVHD. In this study we found higher CD4+ T-cell and consequently higher CD4+CD25highCD127low Treg counts and percentages in patients with cGVHD as compared to patients without cGVHD, but in a logistic regression model Treg counts or percentages were not an independent factor for cGVHD. This cross-sectional analysis of 84 patients after HSCT did not reveal a relation between Treg counts in peripheral blood and the occurrence of cGVHD. We therefore postulate that cGVHD does not occur as a result of peripheral Treg paucity. Similar to this study, Nadal et al. found no differences in CD4+CD25high Treg in peripheral blood in patients with or without cGVHD [18]. Also Clark et al. found elevated percentages and absolute numbers of CD4+CD25high T cells in patients with cGVHD and concluded that cGVHD injury does not occur as a result of Treg deficiency [25]. On the other hand, Zorn et al. found decreased frequencies of circulating CD4+CD25high Treg and lower FoxP3 expression by quantitative PCR in patients with cGVHD [24]. Similarly to the results of this study, Ukena et al. did not find a relation between peripheral CD4+CD25highCD127low Treg and cGVHD in a smaller patient group [20]. This study confirmed the results of Ukena et al. using a similar flow cytometry approach and it generated similar results to those of Nadal et al. [18] and Clark et al. [25], who both used different cytometry approaches. The study by Zorn et al. [24] remains to date the only one which showed an association between decreased Treg and cGVHD. To our knowledge this is the second study that used the CD4+CD25highCD127low staining for Treg enumeration in patients after allogeneic HSCT. This method may be more cost- and time-effective than the use of intracellular techniques.

## 6. Conclusions

In this study we found a positive correlation between CD3+CD4+ T-cell counts and CD4+CD25highCD127low Treg counts and the time after allogeneic HSCT and showed a rapid expansion of peripheral CD3+CD8+ T-effector cells and a delayed recovery of CD3+CD4+ T cells and Treg after allogeneic HSCT. CD3+CD4+ T cells and Treg remain lower than in controls even after 2 years from allogeneic HSCT. The Treg recovery closely follows the CD3+CD4 T-cell recovery. We did not find an impact of peripheral Treg on cGVHD. According to the specific site hypothesis cGVHD takes place in peripheral tissues and therefore Treg and their possible protective effect on cGVHD have to be assessed in target organs [32]. 

## Figures and Tables

**Table 1:** Patient characteristics and transplantation details.

	Patients (*n* = 84)
Age in years (median (range))	46 (19–67)
Gender: male/female	50/34
Diagnosis:	
AML/ALL/MDS	24/11/9
CML/OMF	7/3
CLL/NHL/MM	5/16/6
AA	3
Pretransplant conditioning:	
Myeloablative regimens:	46
12 Gy TBI-cyclophosphamide/12 Gy TBI-etoposide	13/3
Busulphan-cyclophosphamide	8
8 Gy TBI-fludarabine/melphalan-fludarabine	4/16
Other	2
RIC regimens:	38
Fludarabine-cyclophosphamide/fludarabine-busulphan/fludarabine-cyclophosphamide-busulphan	9/3/7
2 Gy-fludarabine	13
Other	6
Donor type: sibling/unrelated	38/46
Stem cell source: bone marrow/peripheral stem cells	2/82
HLA matching (10 antigens): match/mismatch	60/24
CMV serostatus of recipient and donor: both negative/other	22/62
Gender match: match/mismatch	45/39
Blood group match: match/mismatch	47/37
Time after transplantation in months (median (range))	18 (5–120)

CML: chronic myeloid leukaemia; AML: acute myeloid leukaemia; ALL: acute lymphoid leukaemia; NHL: non-Hodgkin's lymphoma; OMF: osteomyelofibrosis; MM: multiple myeloma; CLL: chronic lymphatic leukaemia; MDS: myelodysplastic syndrome; AA: aplastic anaemia; RIC: reduced intensity conditioning; CMV: cytomegalovirus; TBI: total body irradiation; HLA: human leucocyte antigen.

**Table 2:** Univariate analysis of factors with impact on the CD4+CD25highCD127low Treg frequencies and counts in 84 patients after allogeneic HSCT.

		CD4+CD25highCD127low	*P* value	CD4+CD25highCD127low	*P* value
		% of lymphocytes		×10^6^/l	
	*n* = 84				
Time of the Treg investigation from HSCT (days)			0.56		** 0.008**

		Median		Median	
Patient age:					
<45 years	*n* = 36	1.87	0.47	20.95	0.94
>45 years	*n* = 48	2.0		23.1	
Donor type:					
Sibling	*n* = 38	2.14	**0.075**	24.0	**0.17**
Unrelated	*n* = 46	1.8		19.8	
Transplant conditioning:					
Myeloablative	*n* = 46	1.83	0.24	21.75	0.51
RIC	*n* = 38	2.26		20.84	
In vivo T-cell depletion (ATG):					
No ATG	*n* = 30	2.14	0.33	25.65	**0.03**
Use of ATG	*n* = 54	1.81		19.15	
HLA matching:					
Matched	*n* = 60	1.93	0.63	24.5	**0.12**
Mismatched	*n* = 24	1.98		17.87	
Gender matching:					
Match	*n* = 45	1.84	0.63	20.25	0.78
Mismatch	*n* = 39	2.05		22.23	
CMV serostatus:					
Negative/negative	*n* = 22	2.21	** 0.041**	23.86	0.86
Other	*n* = 62	1.81		20.84	
History of acute GVHD:					
None	*n* = 51	2.05	0.44	19.13	0.74
Grade I–IV	*n* = 33	1.91		23.5	
Immunosuppression at time of study:					
No	*n* = 53	1.83	0.46	21.27	0.9
Yes	*n* = 31	2.2		21.05	

CD: cluster of differentiation; Treg: regulatory T cells; HSCT: haematopoietic stem cell transplantation; CMV: cytomegalovirus; GVHD: graft-versus-host disease.

**Table 3:** Univariate analysis for chronic graft versus host disease (cGVHD).

	No cGVHD (*n* = 38)	Limited or extensive cGVHD (*n* = 46)	*P* value
	Median (range)	Median (range)	
Patient age (year)	44.5 (19–63)	46.5 (19–67)	0.17
Time after transplantation (day)	505 (150–2430)	577.5 (164–3606)	0.41
Lymphocytes × 10^6^/l	1383 (99.5–3415.8)	1299 (118–3423.8)	0.68
CD3+CD4+ % of lymphocytes	33.2 (6.3–62.9)	22.6 (3.4–62.9)	**0.02**
CD3+CD4+ × 10^6^/l	249.5 (34–1124.6)	312.4 (32.9–1094)	**0.07**
CD4+CD25highCD127low % of lymphocytes	1.7 (0.08–4.2)	2.2 (0.5–9.5)	**0.026**
CD4+CD25highCD127low × 10^6^/l	17.06 (1.6–79.4)	24.5 (2.96–114.7)	**0.033**

	Number	Number	
Gender (male/female)	24/14	26/20	0.65
Conditioning (myeloablative/RIC)	22/16	24/22	0.66
Donor type (sibling/unrelated)	14/24	24/22	0.19
In vivo T-cell depletion with ATG (no/yes)	10/28	20/26	0.11
HLA match (match/mismatch)	10/28	14/32	0.8
CMV serostatus (negative in both/other)	8/30	14/32	0.46
Gender match (match/mismatch)	22/16	23/23	0.51
Blood group match (match/mismatch)	20/18	28/18	0.5

CD: cluster of differentiation, RIC: reduced intensity conditioning, ATG: antithymocyte globulin, HLA: human leukocyte antigen, and CMV: cytomegalovirus.
